# On Tædium Vitæ

**Published:** 1850-10-01

**Authors:** A. Brierre de Boismont

**Affiliations:** Director of two Lunatic Asyla, Chief Editor of the "Annales Medico-Psychologiques," Knight of the Legion of Honour; formerly Physician to Hospitals in Paris and Warsaw


### ON TÆDIUM VITÆ.

BY MONSIEUR A. BRIERRE DE BOISMONT.*

*Director of two Lunatic Asyla, Chief Editor of the "Annales Medico-Psychologiques," Knight of the Legion of Honour; formerly Physician to Hospitals in Paris and Warsaw.*

PART I.

"THE man who thinks is a depraved animal," wrote J. J. Rousseau; he would have been nearer the truth had he said—"*Un animal qui s'ennuie.*" The paper which I read twelve years ago, at the Institute, "On the Influence of Civilization in Developing Insanity," was freely criticised, but if it were published to-day, would the events which have happened since that period invalidate my conclusions? I think not; and all that I then affirmed about the frequency of insanity in civilized countries, I may with more reason repeat concerning *ennui.*

The earliest description that we have of this *moral malady* is that given by *Seneca,* and is contained in a letter to his friend Seranus. He says—

"The evil which torments us springs, not from the place we live in, but abides within us; we are too weak to bear with anything, we are unable to endure pain, incapable of enjoying pleasure, impatient of everything, and tired of all. How many call out for death, when, after having tried every change, they still experience the same sensations, and cannot rouse a novel feeling. The world, their fortune, and life itself become a burden to them, and, even in the midst of their revellings, they exclaim—'What! the same thing—always the same!'"†

A fatal expression, which we shall find recurring at all epochs, for it is the cry of all

---

* Translated from the original MS. in French.

† Seneca, de Tranquillitate Animi, c. ii. *sub fin.* and Epist. xxiv.

The famous Duke of Queensbury exemplified this state of mind. He had a villa at Richmond, overlooking the Thames, and when any one admired the river, was in the habit of saying—"I wonder what people can find to admire in that river, it is water, and it flows and it flows—it is always the same."—*Trans.*

who prefer reverie to labour, who like emotion better than action, until, to free themselves of the fatigue of existence, they seek a refuge beneath the ponderous and icy tablet of Montaigne. About the time of Seneca, not only was ennui an universal malady, but suicide had become epidemic; nor can we wonder at this, when we reflect on the state of Roman society at that period, as revealed by Juvenal. The mighty empire of the Cæsars was already dead at heart. Some of the outward forms of freedom were still retained, but the spirit had long since fled. There was no security for property or life; no reward for honourable ambition, no incentive to the public service. Foreign auxiliaries alone maintained the glories of the Roman arms; and Grecian sophists quibbled away the simplicity of the Roman law. The pristine faith of Numa had long been lost; virtue had become a scandal, modesty a reproach, and vice the only road to imperial favour. The most depraved lust, and the most refined cruelty; crimes which may not be named, and horrors at which nature revolts, characterized the private entertainments and public amusements of a debauched and degenerate people. The patricians, superior alike in rank and depravity, were also superior in the sense of suffering; many of them, therefore, when exhausted by luxury, overwhelmed by satiety, and grown weary of a shameful life, voluntarily quitted a world which offered them no new thing worth living for.

Christianity deeply modified this state of mind, but its divine precepts did not immediately remove the sadness and disgust which tormented so many men, and ennui then found a refuge in the cloisters. In the writings of the Fathers of the Church, particularly in the three letters of St. Chrysostom to Stagirius, we find an admirable picture of the uneasiness, the inquietude, and indifference which consumed the world in the midst of the most noisy pleasures.

Stagirius was one of those sickly and fickle spirits who think themselves great because they want the qualities which constitute the ordinary and common character of mankind; who create for themselves sorrows and joys apart from all the world, and have the weakness both to envy and despise the calm and simple life of those whom they affect to consider as an inferior race. To deliver himself from his weariness of the world, he had entered a monastery, hoping to find there the tranquillity he sought, but in vain, for he found only what his own discontented spirit had brought with it. "The exterior world," observes Lamartine, in his *Nouvelles Confidences*, "contains nothing comparable with what we see in ourselves."* St. Chrysostom's answer to the complaints of Stagirius is curious, as indicating one of the remedies to his ills, and showing that the young monk, like many other patients, would not bear either the disease or the cure.

"That which most troubles you, Stagirius," says the Saint, "is to see that many men of your acquaintance who were formerly, whilst living in all kinds of pleasure and dissipation, a prey to the demon of melancholy, have been entirely cured when they have married and become the fathers of families, whilst you remain the same, and neither by watching, nor fasting, nor prayer, nor all the austerities of your monastery, are able to obtain any alleviation of your sufferings."†

Thus, as M. St. Marc Girardin observes, it was not for want of distractions and amusements that men fell into that melancholy state, so well expressed by the Greek word, athumia (ἀθυμία). Beautiful slaves, joyous dances, magnificent banquets, the combats of gladiators, the licentious tales of Miletus, voluptuous paintings, and exquisite statuary, were alike of no avail—the *athumia* poisoned all enjoyment. But if, when tired of the search after pleasure, and the despair which accompanied it, they changed their habits, became steady and orderly, and married and had children, then, as if by enchantment, the evil spirit of ennui fled from them. The tranquil pleasures of domestic life dispelled all their miseries; for what vexation can resist the gentle affection of a wife, and the sweet caresses of children. To escape from the nothingness of the present in this world, it is requisite to have a future, and children are the futurity of a family.‡ This peculiarity serves to distinguish the malady of ennui from insanity, for in the latter the family is almost always disregarded, and not unfrequently suspected and disliked.

---

* "Of its own beauty is the mind diseased,
And fevers into false creations," &c.—BYRON.

† St. Chrys. ed. Gaune, t. i. p. 191.

‡ St. Marc Girardin, Cours de Littèrature; du Suicide et de la Haîne de la Vie.

St. Chrysostom next considers what is the kind of melancholy which possesses Stagirius, and demonstrates in the clearest manner that it springs from insubordination and infirmity of soul: capricious afflictions, which any real sorrow would immediately cure, because error cannot stand against truth. After eloquently censuring these fictitious miseries, he analyses them with a keenness of observation not surpassed by any modern moralist, and concludes with this profound maxim—"*The best method to rid oneself of melancholy is not to love it*"—a maxim the force of which we are daily more sensible of. We ought to hate the gnawing cares which vex and worry us, but, as they are bound to our nature by a thousand living fibres, we caress and cherish them with a kind of fondness. We do mean wholly to repudiate sadness of soul, for like all other affliction, God sends it for our good, but we should learn to profit by it; and the only way to profit, is to be sad, not when we suffer, but when we have done ill.

Let us now hear what St. Jerome says upon the subject. "There are some monks, who by the dampness of their cells, by their immoderate fasts, by the weariness of solitude, and by excessive study, fall into a state of extreme despondency, for which they have more need of the remedies of Hippocrates than of our advice. I have seen persons of both sexes, whose brains had become exalted by too much abstinence, especially among those who inhabited cold and humid cells; they knew not what they did, nor how to conduct themselves, nor what to say or conceal."*

After the Fathers we have quoted, evidence becomes more rare, but still from time to time, through the dust of ages, we are enabled to follow the traces of this malady in the monasteries. Thus M. Magnin has discovered, in a MS. tragedy called "Callimachus," written by Krosvita, a monk at Gandersheim, in the tenth century, the subtlety, the sadness, the delirium of soul and sense, and even the tendency to suicide and adultery, which are the almost inseparable attributes of the passion of love in the nineteenth century.†

In the twelfth and thirteenth centuries, a great revolution took place in the minds of men; the relations of society, faith, literature, and the arts, experienced the influence of the crusades. The simplicity of the baronial life, distinguished by its religious sentiment and poverty of ideas, was invaded by a spirit of doubt and inquiry, by a chivalric passion for adventure, and a general relaxation of morals. And then, as M. Bourquelot observes, ennui again seized on entire populations, as it has in our days on a people imbued with a sceptical philosophy, and men and women, monks, nuns, and cavaliers, often experienced a kind of need to terminate their existence. The suicidal mania, confined at first to a few exceptions, sprung up again as a reminiscence of olden times, and invaded all classes of society. Blanche of Castile, the pious mother of St. Louis, on learning the death of her husband, Louis VIII., fell into so profound a despair, that she attempted to destroy herself.‡ The unfortunate Regnault of Boulogne, made prisoner at the Battle of Bovines, when he had lost all hopes of release by the accession of Louis IX. to the throne, killed himself in prison.§ The poems and romances of the twelfth and thirteenth centuries, often contain very touching stories of brave knights and ladyes fair, led by the pangs of love, or the fear of dishonour, to commit suicide. Seneca reveals to us the state of mind of many of his contemporaries, full of weariness and disgust, languishing and discontented, dissatisfied with the past, and without hope in the future, indifferent to what they had done, or what they had to do. Men plunge into solitude without finding the peace of mind they seek; they try all kinds of distraction, they bustle about, they travel from place to place, they supplant one emotion by another, they go from sight to sight, from pleasure to pleasure, ever wishing to fly from themselves, and ever finding themselves tied to the same insufferable companion.||

St. Chrysostom, in his description of *Athumia*, has very well described the utter want of all interest and energy, the depression, or rather annihilation, of spirit which accompanies it; but it is evidently a lesion of volition—the intelligence and capacity for action are unimpaired, but the will to act is wanting. In the account St. Jerome gives of

---

* St. Jerome, Litt. 95, ad Rusticum; 97, ad Demetriadem.

† Revue des Deux Mondes, Nov. 1839.

‡ Chronique de Philippe Monskes, ed. Reiffenberg, t. ii., p. 554.

§ Chron. Alberici, ed. 1698, p. 522.

|| Felix Bourquelot. Biblioth. des Chartes, t. iii., p. 539, 560. Recherches sur les Opinions et la Législation en matière de Mort Volontaire, pendant le moyen âge.

Athumia, the disorder more nearly approaches insanity, for the mind is obviously impaired.

These affections of the intellect are otherwise marked in the *Accedia* of monks, of which Castianus, a writer of the thirteenth century, has left a description; and in the madness and demonolatry of which M. Calmeil has brought to light a great number of examples.

There exists, reports Castianus, a detestable species of melancholy, which, instead of leading men to a greater regularity of conduct, and the correction of their faults, throws them into a miserable condition of despair.*

The ecclesiastical writers frequently notice this moral malady of the monacal societies, to which they give the distinctive name of accedia (ἀκηδία).† This disorder leads directly to suicide; and the number of monks who destroyed themselves under its influence is considerable.‡

The following case is given, among others, by Cæsareus, a Cisterian monk, in his "Discourse on Miracles," composed in the thirteenth century. A nun of advanced age, and exemplary piety, suddenly finds herself troubled by melancholy, and tormented by blasphemous thoughts, by doubts and incredulity; she falls into despair, refuses the sacraments, and fancying herself condemned to eternal torments, and dreading, from the threat of her superior, that her body would be buried in the open fields without funeral rites, throws herself into the Moselle, from which she is with difficulty rescued alive.§

A convert had up to an advanced age, always merited the esteem and commendation of all above him, by the regularity of his conduct, and his rigorous attention to religious observances. But at last he was seized by melancholy; he imagined that his sins were too great to be forgiven—despaired of his salvation; he could no longer pray and was perplexed by overwhelming doubts—and at last drowned himself in a reservoir attached to the monastery.‖ A young nun is seduced by the *magic artifices* of a monk, and, not being able to resist his temptations, wishes to escape from her convent, to indulge her passion. She is prevented, and in despair precipitates herself into a well and so perishes.¶ Baldwin, a monk at Brunswick, having his wits impaired by fasting and watching, hung himself by a cord in the belfry; he is taken down in time, but does not recover his reason.** Castianus also relates that an old monk, named Heron, threw himself into a well and was drowned, but received Christian burial from the hands of his prior, on account of the sanctity of his previous life.††

The authors who relate these suicides, particularly Cæsarius, regard them as so shameful for the monastic institution, that they manifest great hesitation in mentioning them, and abstain from particularizing the places where they happened. Cæsarius seems also to think it injurious for weak-minded persons to be made acquainted with such occurrences.‡‡ He elsewhere expresses himself as follows:—"Perhaps God permits such things to happen, in order that no one, however perfect he may be, should glory in his virtues or good works." The same author seems to think that an immoderate and indiscreet zeal is likely to engender the kind of melancholy called acedia;

---

* Castianus, lib. ix., de Spiritu tristitiæ, ap. Cass. op. omn. ab Alardo Gazœo edita Paris, 1642, p. 193.

† "Accidia est quedam tristitia aggravansque ita deprimit animam hominis ut nihil ei agere libeat, et imo accidia importat quoddam tedium bene operandi. Filiæ accidiæ multæ sunt, quod multis modis per accidiam peccat homo. Ejus autem filiæ sunt hæc. Dilatio, segnities sive pigritia, tepiditas, pusillanimitas, inconstantia, sive imperseverantia et inquietudo corporis, evagatio mentis, ignorantia, ociositas, verbositas sive multiloquium, murmur, taciturnitas mala; indiscretio, gravedo, somnolentia, negligentia, omissio, indevotio, languor, tædium vitæ, impeditio bonorum, impenitentia, desperatio."—Vincent de Beavais, Speculum Morale, fol. Argentinæ, 1476; lib. iii., p. 6.

‡ Histor. Monast. Villariensis, lib. ii., c. 8, in Thes. Anec. D. Martene, t. iii. col. 1368.

§ Cap. xl. Cæsarii monast. Cisteriensis Dialogi miraculorum, Dis. iii., ap. Tessier. Bibl. Cisterc., fol. 1660, vol. i., t. ii., p. 95.

‖ Cap. xli., id. ibid.

¶ Cap. xlii., id. ibid.

** Cap. xlv., id. ibid.

†† Castiani, col. ii., cap. v.

‡‡ Dialog. Mirac., cap. xli.

and in answer to the question of what becomes of the souls of those who destroy themselves? establishes the following distinction:—"If mere sadness and despair, not amounting to frenzy or mental alienation, is the cause of suicide, there can be no doubt that those who commit it are damned. But as for madmen and maniacs who are deprived of reason, there can be no question but that they are saved, in whatever manner they die, provided always that they were at peace with God at the time when their disorders commenced."

These different extracts show that the Church sometimes relaxed her rigours when she saw that there were extenuating circumstances; and that she clearly established the distinction between the moral disorders which result from a perverted disposition of mind, over which the will still has control, and those mental diseases which constitute insanity; they also prove that she knew the dangers of religious exaggeration and contagious incitement.

Sadness, ennui, and disgust of life, maintained and augmented by contemplation, asceticism, and mysticism, and the silent monotony of the cloister, predisposed weak minds, already dreamy and disordered, to receive the social impressions of the age. And as the fear of hell, the dread of devils, and terrors at the expected end of the world, were the predominant ideas during the tenth and eleventh centuries, they were developed in the religious establishments in the form of an epidemic, which has been described as the suicidal monomania of the devil-worshippers.

One wonders in the present day to see condemned criminals evade the hands of justice by self-destruction. The female demonolaters of Northern Germany, says M. Calmeil, were frequently brought before the magistrate, their faces and bodies covered with wounds and bruises. Yielding to a delirious impulse of despair, they struck and injured themselves after the manner of persons possessed; indeed, they themselves avowed that it was Satan who had brought them to that state, and who struck them because he was enraged at their confessions. At last, surrounded on all sides, worn out by internal conflicts, and having in perspective the torture and the stake, they sought a refuge from their sufferings in death, and hanged themselves with shreds of their clothing to the bars of their prison.*

The demonolaters frequently destroyed themselves in disgust at the tyranny of their infernal master, from remorse, and sometimes from the dread of human tribunals. It constantly happened that they hanged themselves, threw themselves into rivers and wells, or made use of some cutting instrument. "One condemned," says Remy, "employed a strip of old linen rag, fastened to a bone stuck in the wall, to hang himself by; and although so close to the ground that his knees almost touched it, nevertheless accomplished his purpose."†

It results from the most careful perusal of old writings, which contain instances of ennui, disgust of life, and tendency to suicide, that such cases were rare in the middle ages, when compared with the 19th century. It is vain to repeat that the question is better understood in these days; that statistical science is in its infancy. We are satisfied that moral facts have always been observed and recorded, and that an exact acquaintance with the predominating ideas, laws, manners, usages, and customs of an age, will enable us to form a precise and accurate estimate of its moral and intellectual condition. Now, all writers on the middle ages agree in stating that, in the 14th, 15th, and 16th centuries, self-murder was considered a crime, and punished as such. This idea, first promulgated by the Christian church, had become a popular opinion, and had passed into the penal law. Doubtless there were suicides in those ages, and M. Bourquelat has recorded some instances; but in spite of the growing freedom of thought, the sentiment of religion, still deeply impressed on the human mind, opposed a powerful barrier, and succeeded in restraining it within narrow limits. However, we do not intend at present to enter into the general question of suicide, but to consider the effect which *tædium vitæ* exercises on that fatal determination.

The intellectual movement of the 16th century naturally produced a host of new opinions; and, joined to the bustling habits of that period, left little leisure for ennui and melancholy; but the examination of matters of faith, and the revival of philosophy, contributed not a little to spread the germs of doubt and scepticism.

The sensualist doctrines of the 18th century, the attacks on religion, and the encouragement given to suicide by many distinguished writers, produced their baneful fruits,

---

* Spranger, in Malleo Maleficorum, p. 166.

† N. Remigius, opere citato, p. 352-3-5-7.—*Vide* Calmeil, De la Folie.

and ennui and disgust of life again seized on the minds of men. Rousseau, in the character of St. Preux, and Goethe in those of Faust and Werther, expressed the sentiments of many of their contemporaries. For although these characters figure in works of fiction, they are nevertheless facts which mark the tendency of the epoch; we intend, therefore, to say a few words about them, and also of René and Raphaël; for St. Preux. Werther, René, and Raphaël, are in reality Rousseau, Goethe, Châteaubriand and Lamartine, great men, who are themselves, in some sort, the microcosm of their times.

Werther is the type of those ardent and exalted characters, who have neither patience nor endurance for common worldly concerns. The spirit of scepticism and doubt has passed as a blight over their youth. When Werther contemplates himself, he finds in his own breast a host of sombre presentiments and ill-defined desires, differing entirely from the realities of active life. This idle melancholy does not calm the passions. He enters from time to time into the business of life, but only for an instant,—for he hastens back to that solitude which is his existence; there it is that he agitates and labours, creating for himself a world apart. He despises any useful occupation, although it may be a means of improving his social position, and is the accomplishment of the divine command, which makes labour the inheritance of mankind. God has placed man on the earth, not to dream away life, but to act; and he has made action the end and aim of all our thoughts and all our sentiments: thus, piety leads to religious worship; love to family cares; the sentiment of the beautiful to the fine arts; the conception of the necessary to useful inventions. The Almighty himself does not rest satisfied with a contemplative existence, but is incessantly occupied in the creation, maintenance, and government of the universe; and man should follow the Divine example, and abstain from useless reveries, which induce an invincible repugnance to exertion, and lead directly to suicide.

We find in Stobœus, a writer of the fifth century, the history of a young man who, being compelled by his father to become a farmer, hanged himself, leaving a letter, in which he stated that agriculture was too monotonous an occupation for him; that it consisted in sowing to reap, and reaping to sow—a weary circle of operations, without end or variety.* This suicide from pride and idleness resembles very many in our own times.

Werther is chiefly wanting in submission to the will of God, and that love of order which makes life easy and pleasant. A child of the 18th century, he has not the firm and simple faith of his forefathers. What is sadly interesting in Werther's case, and it is common among those who do not actually kill themselves, is the internal conflict, the varying progress of his defeat, and the different emotions which intervene betwixt the first dawning of the fatal idea and its final accomplishment. At one time, his soul recals with a fond regret the pleasures of his past existence; at another, he dwells only on the present with an impatient bitterness, which renders everything insupportable, and makes the merest trifle a cause of annoyance. This very impatience is the last struggle of the instinctive love of life with a destructive resolution, and marks the period when the miserable sufferer has neither the will to alter, nor the energy to accomplish his intention. Werther belongs to the school of St. Preux, whom he resembles in the violence of his love. And it is curious to remark the effects which the contradictory influence of Rousseau and Voltaire produced on their contemporaries. Romantic attachments, characterised by a wordy sentimentalism, took the place of the "*bonnes fortunes*," and the frank sensuality of the *Roués*; but it was a change of manners rather than a reformation of morals—there were great words and petty sentiments; very refined language, but very vulgar emotions.

Another point of resemblance is that sensibility which, in spite of the exaltation of expression, is more nearly allied to tenderness of sense than tenderness of soul; and was the meaning of the word tenderness as understood in the 18th century.† This sensibility, half spiritual, and half corporeal, is a poor preservative against the impulse to suicide: "For to be carnally minded is death; but to be spiritually minded is *life and peace*."‡ Thus Werther fell, leaving, as Madame de Staël remarks, the fatal disposition of his mind as a legacy to a generation of visionaries, on whom it produced the most lamentable results.

René, who inaugurated the present century, is the successor of Seranus, Stagirius, and Werther; for, notwithstanding his religious education, doubt is at the bottom of

* J. Stobœus. Sermones: ed Gaisford. Oxford, 1822, t. ii., p. 420.

† St. Marc Girardin.

‡ Rom. chap. viii. v. 6.

his character. This young man, of ardent mind, and boundless imagination, restless and desolate, filled with infinite aspirations towards some unknown and unattainable end—a dreamer, not an actor, and more poet than logician, well personifies a sickly generation of young men, whom the horrors which they had witnessed had disgusted at the world.

At the epoch when René appeared, France had just issued from a revolution which had overthrown the two fundamental pillars of the State, Religion and Royalty. The king, the priest, and the noble, had been swept away in a torrent of blood. Scarcely a family that did not number a victim; hardly a fortune that was not shattered or destroyed; wrecks and breakers everywhere, and nowhere a refuge; all belief was dead, and hope almost. Doubt, despair, hatred, and vengeance, ruled the minds of men. Apostasy, ingratitude, delation, and treachery, in endless combination and variety, showed to what extremes of baseness our passions lead us, and how much refuse lies at the bottom of man's heart. An universal discouragement had succeeded to the faith of preceding ages. When René, a few years later, reappears in his real name, and published his "*Mémoires d'Outre Tombe*," we read at each page the avowal of the ennui which consumes him. Whether as orator, author, ambassador, or minister, he is never happy nor satisfied; every place is wearisome to him, and he seeks relief in frequent changes, until at last, burdened with years and regrets, he sits down sullenly in a corner, and shuts himself up in a disdainful silence. Read Raphaël, who, like René, has revealed in his "*Nouvelles Confidences*" the secret of his name; and you will find all through that weakness, indecision, vagueness, and dreaminess, which belong to all those characters in whom faith is dead. He says:—

"The languor of all things around me was in marvellous consonance with my own languor. It increased it by charming it. I plunged headlong into the abyss of melancholy. But this melancholy was animate—too full of thoughts, impressions, and intimate communications with the infinite—of light and shadow in my soul, for me to wish to withdraw myself from it. A mortal malady—the impression of what is attractive rather than painful, and in which death comes as a voluptuous swooning into the infinite. I was resolved henceforth to yield myself up entirely to it; to sequestrate myself from all society which offered any distraction; or to envelop myself in silence, solitude, and a cold reserve, in the midst of those I should meet there. I wished to make my spiritual isolation a winding-sheet, through which I should see no more of men, but only nature and God."—(p. 31.)

The natural consequences of this disposition of mind were, for Rousseau, Goethe, Châteaubriand, and Raphaël (*Lamartine*), a strong tendency to suicide. Châteaubriand thus relates this event of his life:—

"I am now come to a period when I have need of some strength to confess my infirmity. The man who attempts self-destruction proves, not the force of his character, but the weakness of his nature. I possessed an old fowling-piece, with a lock in so dilapidated a state, that it often went off on the slightest shock. One day, I loaded this piece with three balls, and proceeded to a retired spot, far from the main road: arrived there, I cocked my gun, placed the end of the barrel in my mouth, and then struck the butt against the ground. I repeated this trial several times; still the piece did not explode—the appearance of a gamekeeper suspended my resolution. A fatalist, without knowing or desiring to be one, I supposed that my hour was not yet come, so I put off the execution of my project to another day. If I had killed myself, all concerning me would have perished with me—nothing would have been known of the history which led to the catastrophe; I should have swelled the crowd of nameless unfortunates, for I should not have left myself to be followed by the trace of my troubles, like a wounded man by the track of his blood."*

Raphaël, like Châteaubriand, has also his day of despair:—

"I lashed," says he, "our two bodies together with a cord, which I found in the boat. I then raised her in my arms, which I had kept free, to precipitate her and myself into the sea. At the moment I was making the spring which would have engulphed us both beneath the waves for ever, I felt her head fall like a dead weight over my shoulder, and her body sink down about my knees."†

Thus, at a distance of eighteen centuries, we trace the same sickly condition of mind, masked, indeed, under different forms, but produced by the same passions. The reason is, that in both ages the incentive to activity was equally destroyed. Patriotism

* Mémoires d'Outre Tombe. Presse, 31 Octobre, 1848.

† Raphaël. Paris, 1849. P. 159, &c.

among the ancients, and religion among the moderns, had lost their sway over the heart. A selfish individuality, unchecked by any restraint, distinguished alike both the epochs we have considered, and offers a resemblance of the present calculated to inspire very grave apprehensions. M. Molé was therefore strangely mistaken, when, in replying to the eloquent admission-address of M. Alfred de Vigny, he said so bitterly:—"Nothing resembles the two characters of Chatterton and Kitty Bell, not even those which most remind one of them, as Gilbert, Werther, René, and the whole of that family, sprung from J. J. Rousseau, and all so fascinating to weak and disordered minds. Beyond the eighteenth century, we find no trace of them. They belong exclusively to an effeminate age, to an enervating civilization, in which man, absorbed in self, and compassionating his own destiny, withdraws from his fellowmen, and concentrates his entire existence in a steril and querulous pride." But to us Seranus, Stagirius, seem members of the same family, and ancestors of Werther, René, and the others.

Generally, in those societies which are growing old, says a modern author, the mind has lost the support of faith, and acquired a sad experience of the past, without gaining any confidence in the future; then, to employ the expression of Montesquieu, *the soul, weary of itself*, falls into that pernicious dejection which makes sleep or death the sole thing desirable. Death then presents itself as the only heritage which none can spoil. A false philosophy robs it of its terror, and numerous examples in some measure extenuate the shame; thus the idea of suicide grows, and is propagated.

In terminating this historical summary, we have an important observation to make. The malady of ennui, even when associated with a disposition for suicide, cannot be considered a variety of insanity, unless accompanied by some disorder of the sentiments or intellectual faculties. To wish to make a moral affection an appendix to insanity, would be to fill up a rich mine of observation, and would justify the taunt commonly addressed to psychological physicians, that they ride their hobby in all directions. The ennui of Seranus, Stagirius, Werther, René, Raphaël, &c., is owing more to social than to personal causes; it is a symptom of an old and worn-out civilization—of an epoch of decline in religion, politics, and useful progress. Ennui often leads to madness, but is distinguished from it by well-marked characters; it is a moral malady, which may sometimes call for medical treatment, but which commonly requires for its prevention and cure a different and more powerful mediation.

### Part II.

Up to this point we have examined the question of *ennui* historically—we now propose to study it by means of our own particular researches. These consist of an analysis of 4595 official reports of suicides, and form the basis of an essay on voluntary death, which we have worked at for some years past.

Among the 4595 individuals, the "*procès verbaux*" of whose deaths we have investigated, we find 160 described as having destroyed themselves from weariness of life. Of this number, 40 had been influenced by debility or suffering consequent on disease; 32, by want; 20, by trouble in general; 19, by domestic troubles; 16, by disappointed love; 5, by vanity; 2, by fear; 1, by jealousy; leaving 25 persons whose suicide had been produced by ennui, discouragement, and melancholy. The proportion in the second category becomes much greater if we consult other writings, which give 237 cases (192 males, 45 females.) Thus ennui is ascribed in 138 cases to the ordinary motives already indicated, but in 99 cases it has no other source than itself, and arises from the education, prevailing ideas, temperament, organization, and disposition of the individual.

On arranging these causes in a tabular form, we have the following result:—

### Disgust of Life.

| | |
|---|---|
| From reverie, discouragement, ennui, melancholy, and despair | 99 |
| ,, physical suffering and debility | 40 |
| ,, want | 32 |
| ,, trouble in general | 23 |
| ,, domestic troubles | 19 |
| ,, love | 16 |
| ,, vanity | 5 |
| ,, fear | 2 |
| ,, jealousy | 1 |
| | 237 |

Moral affliction and physical suffering can each produce ennui and disgust of life, but then there are complex elements, and it is necessary to make a distinction. Thus if a husband loses a beloved wife, his life, hitherto full of charms, becomes a burden to him, and he kills himself to escape from his despair. In this case weariness of existence is a secondary cause, the loss of the object of his affections being the starting point of the moral ill. On the other hand, it may happen that reverie, vague thoughts, ennui, melancholy and gloomy ideas, are habitual to an individual, and constitute his natural character; everything saddens, and nothing pleases him, he is always complaining of others, of himself, of everybody and everything. A real additional vexation will be sufficient to drive him headlong into eternity; indeed, some slight exacerbation of his customary state of mind will often suffice to bring about the catastrophe. Here the melancholy state of mind is the primary cause, the casual vexation, the accessory circumstance. An acquired ennui is added to the original ennui.

Esquirol rejected the influence of ennui on self-destruction, and tried to prove that the intellect is always unsound in suicides. He said that fortune's favourites never shorten their own existence from ennui. This assertion of our revered master shows, that he had studied the question, rather as a physician, than as a moralist. A careful observation, in fact, proves that there are dreamy, melancholy natures, constitutionally indolent, but capable of short and sudden displays of energy, who are sometimes seized with such extreme discouragement that they wish for death, and would voluntarily seek it, were they not deterred by moral and religious sentiments. We are the playthings of circumstance, and exposed to so many petty annoyances, which in an evil mood of mind assume gigantic proportions, that "trifles light as air" can at times lead us to the most fatal extremities. How often does it happen that men of superior endowments and considerable self-control, when fallen into a state of nervous irritability, find themselves on the point of yielding to the most insensate rage, ready to smash everything about them, and to commit acts of violence and folly, the mere thought of which would, in their calmer moments, make them blush for very shame?

What observer is there who has not encountered in the midst of those belonging to him, in his family, or among his friends and acquaintances, some one of those restless, romantic, visionary dispositions, impatient of direction or control, to whom any remonstrance is an offence, and each reproach a wound. Their darling scheme is to live alone and free, unshackled by the rules of society or the conventionalities of social life. They are incapable of steady application or any serious work. They live to dream: the reality is odious to them, so discarding the actual world, they delight in an ideal realm. Their imagination feeds on chimeras. Proud, conceited, full of self, vain of their talents, which they are always disposed to overrate, they take a pleasure in living and acting differently from other men, and think they evince their superiority by so doing. They cannot relish domestic joys, and they remember their early youth with feelings of humiliation and regret. As they grow older, their peculiarities exaggerate. If they acquire celebrity, they isolate themselves from their rivals, and allow only their humble admirers to approach. In the midst of a success, which so many envy, they are a prey to a thousand cares. At one moment in a transport of joy; at the next they fall back again into the ennui which oppresses them: nay, their very success is a disappointment, because it comes short of the vast ideal they had formed. '*What! is this all?*' is their cry on obtaining everything they had desired. Sometimes, to engage the activity of their mind, they enter into public life, and give the world the spectacle of their versatility, inconstancy, and adoration of self. In the midst of this fruitless agitation, ennui still possesses them. Their maturity is wasted in endless vacillations, in great and small achievements, in ever-recurring faults and errors, until having lost all public favour they are forced to withdraw and return to solitude. Angry with themselves, and irritated at the world, they pass the remainder of their days in sadness, silence, and disgust—yet happy, thrice happy, if the bitter reminiscence of mourning and desolation has not followed their career.

As for vulgar dreamers, spirits of the second and third rank, who never succeed in escaping from obscurity,—driven back upon themselves, their amusement consists in conjuring up new horizons, which are ever fading from their view, before they can resolve upon making any effort to fix them. If by accident they enter into the world, their projects never go beyond a commencement. Rendered sensitive to the last degree by their contemplative existence, and exhausted by internal conflicts, they have no resolution to face the stern realities of life; the most insignificant obstacle, the slightest contrariety, is sufficient to discourage them. Occupying themselves only by fits and starts

—inconstant, capricious, fidgetty, fantastic,—they are a torment to their family and a burden to their friends. These unappreciated geniuses, believing themselves misunderstood, and saturated with egotism, become more and more sad, morose, and melancholy; everything worries and fatigues them; life seems to them a bitter deception, an insupportable burden, and self-destruction the only cure and escape from so many ills. Well may we ask, is there not some taint of madness in these visionaries? The answer should not be doubtful. We may with reason admit a predisposition, but our social condition, the system of education, the prevailing doctrines and deadening materialism of the age, are sufficient to account for the condition of their minds.

In our times, there is no need to be mad to be stricken at heart with ennui and disgust of life,—when no one is sure of the morrow; when reputation, property, and fortune have no stability; when politicians, both conservative and socialist, commence their writings by this set phrase, "*we march towards the unknown*;" when, on looking around us, we see nothing but ruins; no institution erect, and intelligence driven to shelter itself behind the sword. Do you believe that tranquillity of soul, of which Seneca speaks, to be enjoyed by many? The presentiment of coming ills, would not one say that is general? On seeing whole populations rushing in pursuit of pleasure, does it not seem that they seek to fly from themselves, and turn away from the evils which are at hand? Are they not like the Jews at the siege of Samaria, crying "Let us eat and drink, for to-morrow we die?"

There is an age at which the weariness of life seems closely connected with the development of the sexual instinct. This instinct, but little felt by those whose character or position leads to active exertion, operates powerfully on susceptible and imaginative persons, naturally prone to reverie and solitude. It is about the period of puberty that this species of melancholy first manifests itself. Vague desires and ill-defined sensations now trouble the youthful breast. Resemblances before unseen, and sympathies until now unfelt, have changed the aspect of life.

Then comes a passionate longing for some beloved companion, to share and reciprocate the new-born feeling. In the fancy of Plato, the half-soul seeks its complement through the wide world, in sunny fields and shady woods,—

> "Or forest by slow stream, or pebbly spring,
> Or chasms and watery depths."

Earth, ocean, air, are in turn invoked to yield up the Ideal One, in whom all grace, beauty, affection, and intellect are centred. And this is Love,—more potent and seductive in its vague imagining, than when individualized and devoted to some mortal object. But after a while, when neither flowery spring nor leafy summer bring the enchanting phantasm so eagerly desired, sadness and heaviness fall upon the souls of these dreamers; earth loses its beauty, heaven its light, and existence seems mournful, dark, and gloomy. The direst melancholy attacks them, leading to an intolerable weariness of life and longing for death. This state is common to all gentle and loving natures, of contemplative disposition, and nervous impressionable organization. The ennui and disgust of life which attacks young females, about the epoch of puberty, was remarked on by the ancients.

The susceptibility of impression, so common at this age, may also explain why so many celebrated men have been pursued in youth by the demon of suicide. In his "Mémoires d'Outre Tombe," Châteaubriand has perfectly described the effects of this kind of exaltation. But Love in men of genius, endowed with the poetic temperament, is only a form of the immensity of their ideas. Their life is passed in running after an Ideal, which they never attain, and it is the fatigue and disappointment of this hopeless chase which leads them so early to desire the repose of death. "I composed for myself," says this great writer, "one perfect woman from all the women I had seen. The enchantress who maddened me was a mixture of mysterious sentiments and passions; I placed her on a shrine and adored her. This folly lasted two entire years, during which the faculties of my soul arrived at the supreme point of exaltation."

It is common for *artistes* accustomed to public applause, to fall into the most profound depression and despair if their good fortune deserts them. All who were acquainted with the unfortunate Nourrit, can testify to the goodness, elevation, and sensibility of his character, and yet the participated success of a rival was the beginning of his ills—a fancied hiss his sentence of death.*

---

* Nourrit was for some years first tenor at the Grand Opera, Paris. His health having suffered from too great application to his art, and the incident above mentioned,

The celebrated painter, Gros, was of reserved disposition, and, like all men possessed of great talents, extremely susceptible; but he conversed freely with those who had acquired his confidence. Calling one day upon M. Honoré, who attended him, the conversation naturally fell on his art and his compositions. Gros, after thanking the physician for some just and friendly compliments, said, in a mournful manner, "and yet I get no more commissions." Throughout his visit he remained sad and complaining; and the conversation continuing, he suddenly struck his hand on his head and heart, exclaiming, "Doctor! you, who from your profession know so much of human nature, tell me, do you think I have nothing more here?" This took place a short time before his suicide. At the same interview, Gros related to M. Honoré an anecdote, which shows that, like Michael Angelo, he wasn't to be slighted with impunity. "I had one day encountered the Marshal B.... on the Boulevards; he was an old schoolfellow, but had become an important personage; he noticed me politely, but with a certain air of protection, pressing me at the same time to call upon him. Some days after I went to his house, but he kept me waiting so long time, without seeing me, that I grew tired, and left. Napoleon having commanded me to paint the picture of the 'Plague of Jaffa,' it became necessary for me to take the portraits of the persons who appear in it. As the Marshal B.... was of the number, I wrote to him, requesting him to fix a day for a sitting; however, he took no notice of my note. Feeling hurt at his behaviour, I introduced his figure at full length into the picture, but covered the face with a handkerchief. He was very angry, and complained bitterly to the Emperor; but the handkerchief remains to this day, like the Cardinal among the damned in the 'Last Judgment.'"

Ennui, depression of spirits, and weariness of life, are not found solely among poets, artists, and literary men, but also in men of the most vigorous character. Napoleon is a striking example; and we may also mention Dupuytren.

"There is," we have said, speaking of this great surgeon, "in the lives of illustrious persons, a moment of immense interest; it is that in which, for the first time setting foot in the world, they are about to begin the arduous struggle in which so many fail, and so few succeed. One experiences a strong desire to be made acquainted with the secret of those mysterious years, with their varying alternations of hopes and joys, deception and despair. It is the epoch when the impulse to suicide wrestles with the expectation of future fame, and death, standing face to face with ambition, awaits the signal to drag his victim into the gulf of oblivion. By what efforts did these men succeed in triumphing over the obstacles which surrounded them, and the invincible distrust which attends an unknown name? How did they clear the brazen wall that fate had placed betwixt them and fortune? In the solitude and retirement in which they lived, how came they to find the friend or protector so necessary to them? Undeceive yourself—no one assisted them. What they are, they owe only to themselves and to their own strong character; what their hearts suffered, no one knows. They did not falter on the way; they braved all, and shrunk not from any danger or privation. But at what price have they purchased their brilliant reputation? The sum of their sufferings is really frightful."*

Professor Cruveilhier says that Dupuytren was naturally sad and melancholy, and adds that "from his youth a disgust of life seized hold of him, and a terrible thought, which he always opposed with courage, often troubled his repose."†

Pariset had also had his evil day, and we read in the biographical notice of him, in the *Gazette des Hôpitaux*, that his bosom friend found him one morning busily engaged in making preparation for suicide.

Finally, we may refer to the case of a celebrated journalist, who in a moment of discouragement attempted to blow out his brains, but fortunately for him, merely wounded his shoulder.

In the 4,595 official returns which have formed the basis of our researches, the number of notes, letters, papers, pieces of poetry, left behind by those who quitted life from disgust, ennui, indifference, scepticism, and materialism, amounts to 237. We

---

he was advised to travel. Hearing at Naples of the rising success of his great rival and successor, Duprez, he threw himself from a window, and was killed.—Trans.

* Brierre de Boisment et Marx. Leçons orales de Clinique Chirurgicale faites à l'Hôtel Dieu de Paris, par Dupuytren. Paris, 1839. Six vols. in 8vo. Notice Historique, p. vi. t. 1er.

† Cruveilhier Plutarque Français, T. viii. p. 22.

have divided them into two series: the first, being the most numerous, comprises those in whom spleen and *tædium vitæ* followed some trouble or suffering (*ennui acquis secondaire*); the second includes those suicides in whom reverie and melancholy were constitutional (*ennui original*).

We will select some of the more interesting facts from the two lists; and we will point out, particularly in the second, which is the chief substance of this work, the various shades of primary ennui depicted in these writings.

All kinds of human misery tend to engender ennui and weariness of life; the table we have given indicates several.

"Overwhelmed by years and infirmities," writes a father to his daughters, "past work, wholly dependent upon you, and having in vain tried to get into some hospital, I embrace the opportunity of your absence to rid you of a heavy burden."

"My sufferings," says another, "have made life insupportable to me;" but his neighbours observed that those sufferings did not appear to them sufficient to explain his fatal determination. This recals the remark of Châteaubriand, that "the trials of life are like countries, every one has his own; and to attempt to reduce them to one common type, would be to set aside the peculiar sensibility proper to each individual."

In a third letter we read these lines:—"Although it is two years since I lost my wife, I find it impossible to forget her; my sorrow to-day is as fresh as at first, and ennui follows me everywhere. In vain have I taken a new companion; the memory of my first wife never quits me an instant; so I go to join her in eternity. All that I ask of my family is to bury me by the side of her who, if she had lived, would have prevented all my misfortunes."

On a table by the side of a man who had destroyed himself was found the following reply to a letter from his wife, exhorting him to return to her, as she felt certain that their united labours would assure them both a comfortable existence:—"A prey to a weariness and disgust of life which nothing can overcome, I am moreover unable to support the idea of returning to my native country in the garb of poverty, and to show my fellow-countrymen that my talents, my education, and my labours, have brought me to nothing."

Who is there among us who has not felt the truth of these regrets, and often, in his secret heart, preferred death to such wounds of his self-love?

If we were to report all analogous facts, we should enlarge this extract beyond measure: to deny the influence of ennui in determining suicide, is to deny all evidence. There is even no exaggeration in saying that ennui is the shadow of humanity.

But the aspect in which ennui most interests us is in its simple and primitive form, as a part of the original idiosyncrasy—the ennui of Seranus, Stagirius, Werther, and René. We shall proceed to reveal it in a host of sufferers, assuming, certainly, very different forms, but still the same in substance. No expressions are more common in the manuscripts of suicides than these: Life is a burden to me—it is insupportable —I am tired of it—I feel a horror of the world—ennui consumes my existence, &c. &c. Often it is a feeling of depression and downheartedness which takes away all energy of the will, and all hope in the future.

"My good Friends,—I bid you adieu, for I am resolved to die. I have had so little enjoyment upon earth that I quit it without regret. I have entertained this idea for the last three years. I have always felt that I should never get on by my talents, which are nothing; nor by my wits, which are no better,—so it is not worth the trouble of living to vegetate some thirty or forty years, more or less. Moreover, I find existence too monotonous; no one comprehends me, no heart answers to mine as I wish,—not a single pleasure to make life pass sweetly. I know that I am still young, and what I desire may some day arrive, but I have not the patience to wait, and congratulate myself that I have sufficient courage to rid myself of all future distresses. If I had seen a more brilliant future before me I should perhaps have remained, but I am certain that I shall be easier with five or six feet of earth over my body, than if I was erect. I had always determined never to go beyond thirty-two years of age unless my fortune improved: and now that the period has arrived, my long-fixed resolution does not fail me.

"Excepting my father and mother, and you whom I have always considered my dear friends, I leave nothing behind me that I regret. Never having injured any one, nor committed any action with which my conscience reproaches me, I firmly believe that I shall be happier in another world. The last service I ask you to render me is, to assure yourselves that I am really dead. I have no fear but that I shall finish it, yet it would be very miserable to wake up and find myself between five boards. The way to satisfy yourselves is to open the four veins. One may easily see I do not kill myself in despair, for my writing clearly shows that my hand does not tremble."

One of these *ennuyés* complains of not having received at twenty-three years' of age the education which would have enabled him to obtain a position among the powerful and the rich. He refuses a place which is offered him, as being unworthy of his pretensions, and calls God, his parents, and society to account.

Weariness of life is sometimes due to a constitutional melancholy, which no argument or distraction can remove. Doubtlessly there will be found physicians to contend that this state is the first stage of insane *melancholia;* such is the consequence of a system of over-generalization. But, according to this, all those persons whose nervous and irritable temperaments render the slightest contradiction and most trivial vexation the exciting cause of impatience and disgust of life, should be considered insane. Really, in attempting to prove too much, one proves nothing.

A gentleman, twenty-five years of age, enjoying a fortunate position, and living with his family, by whom he was tenderly cherished, had possessed from infancy a most unhappy disposition, which did not improve with his growth. He was habitually silent, sombre, and melancholy; and when any one inquired the cause of his taciturnity, he avoided answering. He often put some such question as this?—"*Dites-moi, vous ennuyez vous? pour moi, je m'ennuie beaucoup.*" He rarely took any part in the amusements of his friends, and then only at their earnest request. He was always cold, distant, and reserved. During the three weeks which preceded his death, he was observed to employ himself in fashioning a plank of wood, and when asked for what use it was intended, replied that they would see some day.

On the morning of his suicide, he made his usual inquiries about the health of his father, took his breakfast, then returned to his room, and was seen no more alive.

He was found dead in the middle of the very singular preparations which he had made, and which consisted of a fire arm fixed before him, a plank fastened to the wall behind him, to deaden the balls, and a basket of bran beneath him, to receive his blood. He had written several recommendations with a pencil on the walls, and a small casket contained some letters referring to his fatal design.

"I am going to heaven with my mother and Eugène D——, that is, if those who destroy themselves are admitted to the celestial habitations. No one on earth can address a reproach to me touching my honour, probity, and conscience; I die satisfied on these three points. . . . . I regret that my death is useless to my parents and my country."

Upon the panel was written, "The apparatus for my end is completed. . . . . Adieu father, brother, relatives, and friends. . . . . If it be God's will, we shall meet again in the next world. . . . . In my left hand I hold the weapon which is about to send me there. Adieu for ever! . . . . Adieu! Adieu! Adieu! . . . . Pray God for the repose of his soul."

Upon the plank in question he had written, in allusion to the plank itself, and basket of bran,—"By this contrivance the trace of my blood will not stain the floor, and the impression of the four bullets which are about to traverse my body will be marked only on this plank: it is already sufficient that my father's house should be the scene of my death."

He wrote to a painter, who had just taken his likeness,—"When you receive this letter I shall live only in the picture you have so ably executed; my eyes will be veiled, and my image alone can recal to my poor father what they formerly were. . . . . On the point of quitting life, I must set aside the painful thought that I am saying an eternal adieu to my dear relatives. More fortunate than they, nothing but the separation is terrible to me; my resolve accomplished, all will be annihilated—imagination, organs—and I shall be inaccessible to all temptations. But that is not enough; egotism never had a place in my heart, and the intoxicating anticipation of the repose that I shall enjoy in death does not blind me to the afflicting position in which I leave my father and brothers. May they find in the features so faithfully copied by you some consolation for their cruel sorrow.

"By ten o'clock to-morrow morning I shall have yielded up my soul to God, unless some unforeseen obstacle independent of my will prevents it."

In his letter to his father he describes the ennui which had so long consumed him, and which he found it impossible any longer to resist; "for in the conflict," he adds, "I should certainly become a prey to insanity."

The suicidal idea is sometimes incessantly present for al ong period, without those whom it pursues having a real motive for it. Nothing amuses or interests them, and mere existence becomes a burden too heavy to be borne. "This pistol," writes one of these unfortunates, "is destined for me alone, it will injure me only. During the past six years this idea has never left me. I always carry the weapon of destruction about with me; but of late especially I am assailed by mournful presentiments and thoughts of death. In short, I believe the moment near at hand in which I shall put an end to so miserable a life."

In the words and writings of those who destroy themselves, we can often trace their characters and habits, and the influences to which they have been subjected. These commit suicide because they are tired of servitude; those take their departure without leave because they have found no one deserving of their praise. They do not wish for any one to accompany their corpse,—the common grave and pauper's cart is all they ask for.

Many of these miserable creatures are the refuse of the capital; poor wretches abandoned from their earliest youth, and left to wander about the streets, to live as they can. Attaching but little value to life, they quit it when they can no longer satisfy their coarse and sensual wants.

"Punishment, privations, obedience," cries a soldier, "I will have no more of it; let them pick up my corpse and bury it, that is the only service I ask of them. The thought of God has never engaged me, and I do not believe in another life."

There are some who complain of being strangers to those about them; of the miserable fate which pursues them; of finding no consolation; of being unable to support poverty and misfortune; of being tormented by imaginary ills.

Sometimes the motives which impel to suicide are found in the painful reflections which accompany a daily struggle with want and the petty vexations of life, augmented by the desire for enjoyment which cannot be procured.

The invincible repugnance which some experience for every kind of occupation, renders their tiresome life a weary load; everything disgusts them. One of these individuals complains to his sisters of having always to work, and of having no time to divert himself. This pariah, however, easily earns his six francs a-day; but he belongs to that numerous class of mechanics, who, without talent or education, idle and dissipated, are always discontented with their lot, and wish to eat, drink, and amuse themselves, without making any exertion.

The pangs of remorse which follow a debauched and worthless life, inevitably lead to ennui and disgust. A man of this stamp states that he is tired of such an existence. "I am engaged," says he, "to fight to-day with the father of a family, whom I have cruelly offended. If I kill him I feel that I shall be eternally tormented by the bitterest remorse; it is, therefore, better for him and for me, to have done with it at once."

Many young persons are unable to bear the least contradiction without giving way to ill-temper, and all the extravagance of an ill-regulated imagination. Their minds, nourished only with frivolous reading, and unused to serious or useful exercise, delight in paradox and exaggeration, so that, when they meet with the least resistance to their caprices, they become irritated, curse their existence, and threaten to end it.

The same apostrophies to misfortune are repeated in numerous letters. A young man writes: "Life had become a load too heavy for me, and I had not strength to support it any longer; do not pity me, for I was too wretched." Another exclaims, "Since the age of fifteen, I have always been unfortunate; one thing alone attached me to existence—my love for you. If you have often found me cold, it was that my ill health would have rendered your life more miserable; it was preferable to put an end to my sufferings; I have had the strength. That you may still be happy is my final prayer."

When this difficulty of living is carried to its extreme degree, the strongest sentiments natural to man are unable to restrain it. Many letters are conceived in terms like these:—"Life is become insupportable to me; I am resolved to finish it. Accept my farewell. I confide my daughter to you: be a father to her; take care of her; watch over her conduct, and treat her as though she were your own." Some individuals supplicate good and charitable souls to take charge of their children.

Weariness of life persists in all ages. "I am past sixty," writes a tradesman, "and finish my career. I have remained long enough upon earth; alone, without relations or friends, I march off, without drum or trumpet, to travel in the comet." His letter concludes thus:—"Let them carry me at once to the cemetery, in the pauper hearse; I wish no one to follow my corpse."

Among those who commit self-destruction, some surround themselves with objects calculated to strengthen their idea. The following works, among others, have been found open beside their bodies, "Young's Night Thoughts," "Trial of Alibaud," "Reflections of Madame de Staël," "Werther," &c. In antiquity, Cato of Utica read the "Phædrus" of Plato, before piercing himself with his sword. It is by no means rare for individuals who kill themselves from ennui, to record their reflections at the fatal moment, and even describe, with extreme sang-froid, the reflections suggested by the mode of their death. One of the most curious of these facts is that of a man who was able, during one hour and five minutes, to follow the progress of asphyxia.

"I am tired," he writes, "of continually wrestling with ennui, sadness, and misfortune, without ever getting the upper hand; this does not refer to my affairs, for I am not in debt, and have money owing to me. But the malice of certain persons who endeavour, by every means, to compromise my reputation gives me more uneasiness than anything that I could be called on to endure. If they are accessible to pity, they will restore my memory after having calumniated it: I forgive them, but I doubt whether those who are so cowardly as to injure in secret, durst confess their wrongs in public. Lately, a subaltern of the 2nd regiment of Artillery, delivered himself from his troubles by lighting and blowing with his mouth the charcoal that was to destroy his life. I do not pretend to show more courage, (or cowardice, according as one calls it,) but I intend to employ the few minutes that remain to me in describing the sensations attendant on self-asphyxiation, and the duration of the sufferings. If that is of any utility, my death will have been in some degree serviceable. If I come to an abrupt conclusion, it will not be owing to any pusillanimity on my part, but to the inability to continue; or that I have preferred to accelerate the catastrophe.

"7h. 31m. P.M.—My ill fortune pursues me: I am four hours and a quarter behind-hand in the execution of my project. Some troublesome persons came to the door, and I was obliged to open it for fear of exciting their suspicions.

"7h. 45m. P.M.—All is prepared. My pulse beats sixty to sixty-one strokes a minute. I light a lamp and a candle to see which of the two will go out first. I ask indulgence of the *savans* if I do not employ the scientific terms. I wait until eight o'clock to light the charcoal.

"7h. 55m.—The pulse beats eighty strokes a minute.

"7h. 58m.—Ninety pulsations and upwards.

"8h.—I light the charcoal.

"8h. 3m.—The fire is out, and I am obliged to re-light it. Slight headache.

"8h. 9m.—Eighty-five pulsations a minute; the chimney of the stove has just fallen.

"8h. 13m.—The headache increases: the room is full of smoke, which gets into my throat. Pricking sensation in the eyes: a feeling of constriction in the neck: sixty-five pulsations.

"8h. 20m.—Combustion proceeding actively,

"8h. 22m.—I have smelt at some smelling salts, but it has done me more harm than good. My eyes are becoming watery.

"8h. 23m.—A pricking feeling in the nostrils. I am beginning to suffer.

"8h. 25m.—I drink a little water. I can hardly breathe. I stop my nostrils with my handkerchief.

"8h. 32m.—I feel better with my nose stopped up; the pulse beats sixty-three.

"8h. 33m.—The lights are losing their brightness: I upset the water, which I have great pleasure in drinking.

"8h. 35m.—The headache augments. I feel a shivering throughout all my frame.

"8h. 40m.—The light of the candle grows more feeble than that of the lamp. Only one furnace burns well. The stove does not burn at all.

"8h. 42m.—Headache more violent. The lamp continues to burn better; in truth, I turn it up from time to time. The stove is burning up.

"8h. 49.—When I keep my nostrils closed my eyes water more freely. The candle gives only a glimmering light. My ears tingle.

"8h. 21m.—The candle is almost extinguished; the lamp still burns. I feel sick, and should like some water.

"8h. 53m.—I suffer all over. I stuff my nostrils more tightly.

"8h. 54m.—The candle is extinguished; the lamp still burns.

"8h. 56m.—Eighty-one pulsations. My head feels very heavy, and I can hardly write. The furnaces are well alight.

"8h. 58m.—My strength is failing; if I had water I would take some. The lamp is still alight. The headache increases; the oppression is redoubled.

"9h.—I make a last effort. I have taken some water, but all is over. I do not go straight off; I suffer horribly. The lamp is still burning.

"9h. 1m.—I feel a little better. I have taken some more water. The lamp grows dim. A delirium seizes me.

"9h. 5m.—The . . . . ."

In young persons inclined to melancholy, isolation and solitude can only augment this disposition. One of these poor neglected ones thus paints the state of his feelings:—

> "*Jamais d'enfant! jamais d'épouse!*

> *Nul cœur près du mien n'a battu!*

> *Jamais une bouche jalouse*

> *Ne m'a demandé, D'où viens-tu?*"

"Never a child for me! never a wife! No heart has beaten near mine! Never has a jealous mouth inquired of me, Whence comest thou?"

The impossibility of satisfying their inclinations, the being deprived of pleasures to which their age inclines them, are with some young persons the causes of suicide.

"I adore women," writes one of these, "and I cannot have them. I like the theatres, horses, and good cheer; but my poverty is an invincible obstacle to my desires. Such a struggle is intolerable, and renders existence a charge to me. To live in privation is beyond my strength; ennui and despair are slowly killing me; I prefer, therefore, to finish at once."

There are some men who, full of love for their fellow-men, seek by every means in their power to ameliorate their condition, attacking abuses and those who profit by them, retreating from no obstacle or danger. Most of these men die in the midst of their work, in tears and misery. Witness Chervin and many others. If they are courageous, talented, and persevering, and consequently dangerous, they are circumvented, or bribery is resorted to; but if *ruse* and intrigue are powerless against them, their enemies form a league. The conspiration of silence is commenced, and a thousand injurious and calumnious reports are circulated concerning them. Steeped in misery and humiliation, the unfortunate enthusiast has no longer any faith in his mission; despair seizes on him, and he disappears from the scene.

A few years ago, a young compositor, who had probed the wounds of the social body, published a book in aid of his fellow-workmen; it contained some ideas which were well received, but never put in action. Disappointment fell on the soul of the young reformer; and being convinced of the uselessness of his efforts, he resolved to put an end to his days. He explained his motive in the following letter:—

"I freely forgive all who have injured me, and I beg the forgiveness of all whom I may have injured.

"I die convinced that I have written a book useful to the working classes; I still hope that it may conduce to their emancipation, and lead to the institution of wardens, as I demand.

"I am sure, that in the interests of order, in the interests of society, and I speak after having studied the question deeply, and with a personal knowledge and experience of the working classes, there are two methods of proceeding—that which I advocate is the most favourable to the workmen; it is that which will free them the more surely, and give them their place in the social scale. If the government adopts it, material revolutions seem to me impossible.*

"I thank the gentlemen of the press who have noticed my work. I recommend the workmen to make use of that way of publishing their grievances; which will always remain open to them so long as they behave with moderation, and which they may be assured is the only one which will emancipate them.

"If it be asked why I destroy my life, here is the reason:—In the present condition of society the more independent a working man is, the better for him.

"If he has any affection for his family, and desires their welfare, he experiences a thousand sufferings; but if he really loves his fellow-men, has the good of society and the happiness of mankind at heart; if he devotes his abilities, and spends his time for them, his end will be like mine.

---

* This was written five years before the Revolution of February.

"P.S.—I wished to do something for the aged workmen; a Royal Asylum for Industrial Invalids is urgently demanded."

Ennui in the female sex presents no peculiar characters. "For some time past," writes a young woman, "I am worn out with sad ideas and thoughts of death. Fatal presentiments torment my imagination. In short, I may say that the moment is not far off when I think I shall put an end to my miserable existence."

It appears, however, a positive fact, that primitive ennui, from pure disgust of life, is less common among women than men, which is to be attributed to their religious principles, to their love for their family, and particularly for their children, to the difference of their passions, and their facility for occupying themselves.

The existence of ennui, as a moral malady, is sufficiently proved by history and observation: its frequency is placed beyond doubt. It is especially in epochs of general indifference, of doubt, and of individualism, that it commits its ravages. The best method of combating it is to oppose to it a lively faith, serious convictions, and some useful occupation. But in the absence of these powerful palladia, for the present hidden, we must be satisfied with what physicians call the treatment of symptoms.

Three means are principally indicated by St. John Chrysostom in his letter to Stagirius; and as they appear to us the best in such circumstances, we cannot do better than to repeat them.

The first is, not to indulge the melancholy which arises from ennui; the second, to have a family. "It is not good for man to live alone," says a Christian writer; with a wife and family, solitude is impossible; one must be active, provident, persevering, always looking forward; for it requires many years to bring up one's children, and to establish them in the world. The third, which is not the least important, is to have a profession. Labour is the ordinance of God. Idleness never entered into the views of Providence, and it will become more and more impossible in the coming time.

By proposing to themselves, as early as possible, some attainable end, and by pursuing it with activity, many young men will succeed in vanquishing their melancholy, and become useful members of society. Something more, however, is requisite to produce a general result—the restoration of religious faith; to effect this, an universal effort is necessary, and to this should be directed the talents and energies of all the ministers who march under the banner of Christ.

### CONCLUSION.

The numerous facts contained in this essay do not allow us to doubt that suicide often results from ennui and disgust at life.

This first point established, we must acknowledge that the suicides comprised in this category form two sub-divisions. In the first are included those cases—and they are the most numerous—where suicide is the consequence of ennui and weariness of life, produced by moral or physical suffering. In the second are placed those cases in which self-destruction is owing to constitutional melancholy, or natural gloominess of disposition. In the one class the weariness of life is secondary; in the other it is primitive.

Weariness of life is often produced by an abuse of revery, by the predominance of thought over action; in a word, by the want of any active occupation. This state of mind is, above all, common at epochs of general indifference, religious and political. The disposition to ennui is also due to the excitation of the passions about the period of puberty, to the susceptibility of the imagination, and the melancholy often engendered by it.

Wounded self-love among *artistes*, the failure of their various schemes among the ardent and energetic, the nature of the ideas and writings of the time, often conduct to disgust of life.

An exaggerated sentiment of pride, an extreme susceptibility to disappointment, tend in many young persons to produce an abhorrence of exertion and weariness of life.

Generous, humane, and exalted natures, devoted to the amelioration of the ills of life, may also sometimes become weary of their own existence, upon seeing the inutility of their efforts.

The natural melancholic humour causes suicide, but does not constitute a form of insanity, except it is accompanied by some disorder of sensation or intellect.

Weariness of existence manifests itself at all stages of life—in the young man and in the old. The only treatment that can successfully combat with this grave malady, is the constant pursuit of some active occupation. When complicated with mental alienation, it requires special methods.

In fine, and this conclusion is the *résumé* of our labours, *intolerance of life is often a cause of suicide without being attended by any symptom of insanity.*

